# Supplementary material for: Conditional chemoconnectomics (cCCTomics) as a strategy for efficient and conditional targeting of chemical transmission
Source: eLife. 2024 Apr 30;12:RP91927. doi: 10.7554/eLife.91927 (PMC11060718; doi:10.7554/eLife.91927)
Supplement: Figure 3—source data 3. [file elife-91927-fig3-data3.zip › Figure 3 source data3 part 1.zip!/Figure 3-Source data 3-part1/1063f3-1-CRISPResso_Report_kboyr13q.zip!/CRISPResso_on_1063f3_4/3b.Insertion_deletion_substitutions_size_hist.pdf]

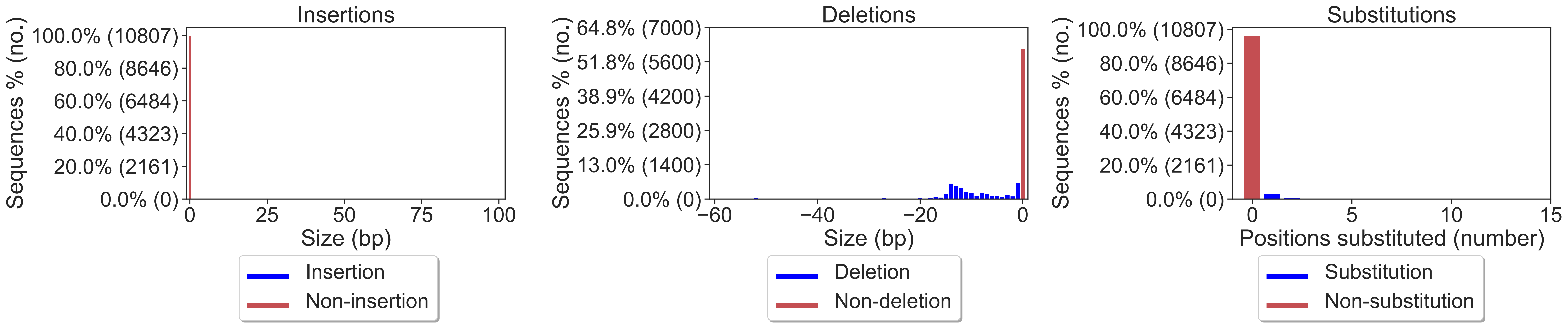
Insertions
Sequences % (no.)
100.0% (10807)
80.0% (8646)
60.0% (6484)
40.0% (4323)
20.0% (2161)
0.0% (0)
0
25
50
75
100
Size (bp)
Insertion
Non-insertion
Deletions
Sequences % (no.)
64.8% (7000)
51.8% (5600)
38.9% (4200)
25.9% (2800)
13.0% (1400)
0.0% (0)
−60
−40
−20
0
Size (bp)
Deletion
Non-deletion
Substitutions
Sequences % (no.)
100.0% (10807)
80.0% (8646)
60.0% (6484)
40.0% (4323)
20.0% (2161)
0.0% (0)
0
5
10
15
Positions substituted (number)
Substitution
Non-substitution
